# Activity-based measures of landscape fragmentation

**DOI:** 10.1007/s10980-024-01987-w

**Published:** 2024-11-16

**Authors:** Barbara Kerr, Tarmo K. Remmel

**Affiliations:** Faculty of Environmental and Urban Change, York University, Toronto, ON M3J 1P3 Canada

**Keywords:** Fragmentation, Activity-based metrics, Least-cost-path analysis, Simulation, Composition, Configuration

## Abstract

**Context:**

Landscape fragmentation, which has demonstrated links to habitat loss, increased isolation, a loss of connectivity, and decreased biodiversity, is difficult to quantify. Traditional pattern-based approaches to measuring fragmentation use landscape metrics to quantify aspects of the composition or configuration of landscapes.

**Objective:**

The objective of this study was to examine the relative improvements of an alternative activity-based approach using the cost of traversing a landscape as a proxy for fragmentation and compare it with the traditional approach.

**Methods:**

One thousand binary landscapes varying in composition and configuration were simulated, and least-cost path analysis provided the data to calculate the activity-based metrics, which were compared with computed traditional pattern-based metrics.

**Results:**

Activity-based fragmentation assessments were sensitive to levels of landscape fragmentation, but offered improvements over exiting pattern-based methods in that some metrics varied monotonically across the spectrum of landscape configurations and thus makes their interpretation more holistically meaningful.

**Conclusions:**

This study provides a modular conceptual framework for assessing fragmentation using activity-based metrics that offer functional improvements over existing pattern-based approaches. While we present a focused theoretical implementation, the process to be measured and the scale of observation can be altered to suit specific user requirements, ecosystems, or species of interest.

## Introduction

Landscapes comprise natural and anthropogenic features that are continually evolving due to interacting processes that alter their composition and configuration. This re-shaping of landscapes can result in fragmentation, where tracts of land cover are portioned into smaller, more isolated areas (Forman and Godron 1986). While fragmentation is a landscape-level process, the term can also characterize the instantaneous structural state, or degree of fragmentation, for a landscape (Forman 1995; Jaeger 2000; Fahrig 2003; McGarigal et al. 2005). Thus, fragmentation can be regarded as a sequence of interacting spatial processes (i.e., perforation, incision, dissection, dissipation, shrinkage, and attrition), that transform land cover from one type to another to create complex landscape patterns through time (Jaeger 2000), that can at any point be at a specified level of fragmentation.

Fragmentation typically increases the edge length of a land cover type that can negatively alter the habitat required by some (often native) species and thus leading to local extinctions or the reduction of biodiversity (Pfeifer et al. 2017), though biodiversity increases have been observed, the increase is often due to undesirable species assemblages (With 2002). Ecosystems affected by fragmentation are characterized by changing conditions within the new and smaller areas where resources and their flows within and among landscape patches will be affected (Rutledge 2003). Landscape fragmentation also has demonstrated links to habitat loss, increased isolation, and reduced connectivity, each of which can further accentuate decreases to local biodiversity (Neel et al. 2004; Kupfer and Franklin 2009; Haddad et al. 2015; Ribeiro et al. 2017). Adverse affects to species diversity through fragmentation (Walz and Syrbe 2013) by increasing vulnerability to invasive species (With 2002), specifically when driven by anthropogenic activities, can alter ecological processes and lead to further habitat decline and loss of species (Hobbs 1993).

The effects arising from fragmentation vary widely according to the landscape altering processes involved and the organism(s) of interest. Different responses to fragmentation are typically observed between generalist and specialist species (Bogaert 2003; Laurance 2008) that have varying requirements for establishing themselves in an area. Therefore, quantifying the fragmentation patterns to predict its effects and apply conservation or management guidelines remains important, yet there exists a lack of agreement on how to measure landscape fragmentation (Bogaert 2003; Kupfer 2012; Lausch et al. 2015). We propose to measure the effects of landscape fragmentation processes on the “activity” of a theoretical organism utilizing a landscape. In this way, if significant changes to an organism’s activity on a landscape are found, that can become a proxy measurement of landscape fragmentation.

There has been debate over the role of fragmentation relative to habitat loss in explaining decreases in biodiversity and the degradation of ecosystems (Fahrig et al. 2022). However, in the past, many studies have not differentiated between habitat loss and habitat fragmentation (Fahrig 2003; Hadley and Betts 2016). For example, if a habitat patch is removed there is a reduction in habitat amount: loss occurs but there is not an increase in fragmentation. Similarly, if a large patch is reduced in size, there is habitat loss but not an increase in fragmentation (Fahrig 2019). Although habitat loss is widely considered to have been a major contributor to a loss of species richness and diversity (Jaeger 2000; Fahrig 2003; Fletcher et al. 2018), observational and experimental studies have demonstrated that fragmentation also has multiple simultaneous effects that are interconnected in complex ways and can operate over long time scales (Haddad et al. 2015).

Given the potential impact of landscape fragmentation on a wide range of ecological processes, it is important to quantify fragmentation (Fahrig 2017), though this can be challenging (Tischendorf and Fahrig 2000; Wickham and Riitters 2019). Quantification is a prerequisite to the study of landscape function (Kupfer 2012) and is used to quantify how landscapes change over time (Turner and Gardner 2015), especially in response to different types of disturbances or land-use pressures (Hesselbarth et al. 2019).

The patch-matrix model provided an early approach to standardizing an approach for systematically characterizing landscapes and their structure (Forman and Godron 1986), relying on the partitioning of landscapes into a mosaic of homogenous areas, connecting corridors, and the background. Changes to these elements could be linked to fragmentation. During the past several decades, the description and analysis of landscape patterns flourished in landscape ecology (Costanza et al. 2019), with hundreds of landscape metrics being developed, implemented, tested, and compared (Baker and Cai 1992; McGarigal and Marks 1995; Haines-Young and Chopping 1996; Uuemaa et al. 2009). These developments have spurred extensive landscape ecological research (Wiens 2008), including those focused on landscape fragmentation (Driscoll et al. 2021).

It is difficult to compare fragmentation studies because authors have not only used different definitions for fragmentation, they have measured fragmentation in various ways and at varying spatial scales (Fahrig 2003). The large number of metrics available also make it difficult to choose an appropriate metric (Neel et al. 2004) particularly at an appropriate scale (Gustafson 1998; Riitters 2019). Furthermore, the large number of available metrics means that there are also many redundancies (Uuemaa et al. 2009) marked by correlations among them (Riitters et al. 1995; Cushman et al. 2008). Many metrics are also highly correlated with class abundance, which makes it difficult to determine whether it is the amount of a specific land cover class or its spatial configuration that accounts for measured values (Remmel and Csillag 2003; Wang et al. 2014) meaning that results are not always intuitive. There is great evidence that pattern-based landscape metric values will vary with spatial resolution (Qi and Wu 1996) and study area extent (Saura and Martinez-Millan 2001; Wu 2004; Šímová and Gdulová 2012), making statistical testing and comparison of such results challenging (Remmel and Csillag 2003; Remmel and Fortin 2013).

In the organism-centred perspective in landscape ecology, the focus is on a particular target habitat according to the species under study and thus, the area of interest depends on the spatial pattern that is meaningful for that species (Antrop 2021). Traditional pattern-based approaches to measuring the fragmentation of landscapes uses metrics that rely on measuring the relationships resulting from the composition and configuration of landscape elements. We pose an alternative approach rooted in measuring alterations to simulated activities that play out on landscapes. Our hypothesis is that landscapes that are increasingly fragmented will affect the simulated activity and thus act as a proxy for characterizing fragmentation. Thus, the absolute composition and configuration are not measured to mathematically produce a fragmentation metric, but the impact of the landscape pattern should influence the proxy measurements related to the activity and thus build a connection between the observed landscape spatial pattern and the impact it has on the simulated activity. The influence can be attributed to the degree of fragmentation. The long history of landscape ecology emphasizes the links between ecological patterns and processes (Turner 1989; Gustafson 1998); thus quantifying fragmentation may provide insights to why landscapes are in their observed states. In this study, we consider landscapes discretized onto a regular grid, where cells can take on one of two values, resulting in a binary (presence/absence) representation for a land cover class of interest.

This study proposes new ways to quantify fragmentation and to test for incremental improvements over existing methods. The goal of this study was to develop and test activity-based fragmentation quantification methods and to then compare those to existing pattern-based fragmentation quantification methods. We focus on answering three research questions: (1) is an activity-based quantification of landscape fragmentation more sensitive to landscape fragmentation than a pattern-based quantification, (2) how sensitive are the fragmentation metrics to landscape simulation parameters, and (3) is there information from activity-based metrics that is not provided by pattern-based metrics?

## Methods

Manipulating and controlling large numbers of replicate real landscapes is not operationally realistic. Therefore, we simulated 1000 binary landscapes with 256^2^ pixels each using a Conditional Autoregressive (CAR) model (Remmel and Csillag 2003) which was parameterized with a proxy measure for spatial autocorrelation and then density sliced to produce the desired binary proportion of land cover classes. This approach, implemented in the R environment (R Core Team 2021), maintained a constant spatial resolution and scale, varying only the composition and configuration of the 1000 binary landscapes. The two simulated land cover classes were hypothetically constructed but could be imagined as representing cells that are easy or difficult to traverse by some species of animal. This level of traversing difficulty is a friction or cost surface (Dutta et al. 2022) that could be defined in any way to represent the environment and/or species of interest. Our convention is that white cells are those with a low “cost” to traverse and black cells are those with a relatively higher “cost”.

Our simulations were controlled by two key parameters: (1) class proportion (*c*) that varied from 1 to 99% white cells and was randomly selected from a uniform distribution for each landscape, and (2) a proxy for spatial autocorrelation (*ρ*) that varied from 0.00000 to 0.2499999, also randomly selected from a uniform distribution for each landscape. The values for *ρ*, when multiplied by 4, return a value like the typical value expected by Moran’s *I*. Each simulated landscape was saved along with the parameters used to produce it (*c* and *ρ*).

For each simulated landscape we computed a suite of 25 commonly used pattern-based fragmentation metrics (Table 1) implemented using the ‘*landscapemetrics*’ package for R (Hesselbarth et al. 2019); please refer to this reference for definitions regarding individual metrics and their formulae. The 25 results for each of the 1000 simulated landscapes were subsequently linked to each respective landscape. While distance and area are provided in specific units (e.g., meters and hectares), these values are assessed in relative rather than absolute terms due to our study design maintaining an arbitrary and constant spatial resolution and extent throughout.

**Table 1:** Selected pattern-based fragmentation metrics used

Area and edge symbol	Name
area_cv	Coefficient of variation of patch area
area_mn	Mean patch size
area_sd	Standard deviation of patch area
cai_cv	Co-efficint of variation of core area index
cai_mn	Mean of core area index
cai_sd	Standard deviation of core area index
dcad	Disjunct core area
ed	Edge density
lpi	Largest patch index
te	Total edge

**Shape symbol**	**Name**
shape_cv	Covariance of variation of shape index
shape_mn	Mean shape index
shape_sd	Standard deviation of shape index
para_cv	Coefficient of variation of perimeter area ratio
para_mn	Mean perimeter area ratio
para_sd	Standard deviation perimeter area ratio

**Aggregation symbol**	**Name**
cohesion	Patch cohesion index
division	Division
enn_cv	Coefficient of variation. of Euclidean nearest-neighbour distance
enn_mn	Mean of Euclidean nearest-neighbor distance
enn_sd	Standard deviation of Euclidean nearest-neighbor distance
mesh	Effective mesh size
np	Number of patches
pd	Patch density
split	Splitting index

In addition to the 25 pattern-based fragmentation metrics, we also propose 5 new activity-based fragmentation metrics, and compute those for each landscape as well. While what constitutes an “activity” can vary (this is modular and modifiable within our code), we opted to compute a relatively simple to implement least cost path (LCP) from the west margin of our landscape to the east margin. The single direction of our activity implementation reflects the stationarity of our landscape simulator, but this remains flexible and modifiable as required, should the underlying landscapes require something omnidirectional instead. Our assumption was that changes to the relative proportion of the landscape classes and how the classes are arranged, would affect movement across each landscape. Thus, trips across a landscape would be more, or less, costly depending on the underlying spatial structure of the landscape classes. When a landscape is altered by fragmentation, costs of traversing the landscape were predicted to increase.

**Fig. 1:**
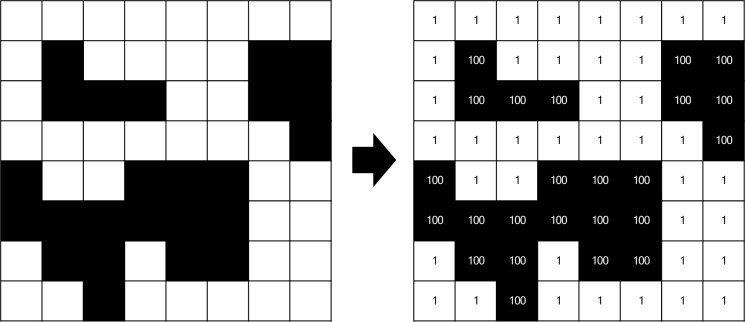
Subset of a binary cost surface raster derived from a simulated landscape with white cells representing 1 “cost unit” and black cells representing 100 “cost units”

We arbitrarily set the cost to traverse white cells as 1 “cost unit” relative to the cost to traverse black cells as 100 “cost units” (Fig. 1). Thus, the lowest cost to traverse a landscape (a direct horizontal line) would be 256 if all cells in that row are white, or maximally 25,600 if all the cells in the row were black. To capture the inherent variability of each landscape, we computed 100 LCP for each landscape, each with randomized starting and end points along the identified landscape margins. We used the R package ‘*leastcostpath*’ (Lewis 2022) to calculate our LCP on each landscape, using rook’s connectivity (raster cells are considered connected or neighbouring if their edges touch). Our simulated binary landscapes serve as the cost surface required by the algorithm and are the spatial representations of the cost of movement across each cell comprising a landscape (Dutta et al. 2022). For each LCP, we computed 5 new activity-based fragmentation metrics: (1) number of steps, (2) cumulative cost to traverse the landscape from west to east, (3) cost-per-step, (4) Manhattan distance, and (5) ratio of the number of steps relative to the Manhattan distance. These results were also linked to their respective simulated landscape such that for any landscape, the simulation parameters, pattern-based fragmentation metrics, and the new activity-based fragmentation metrics could be compared.

**Fig. 2:**
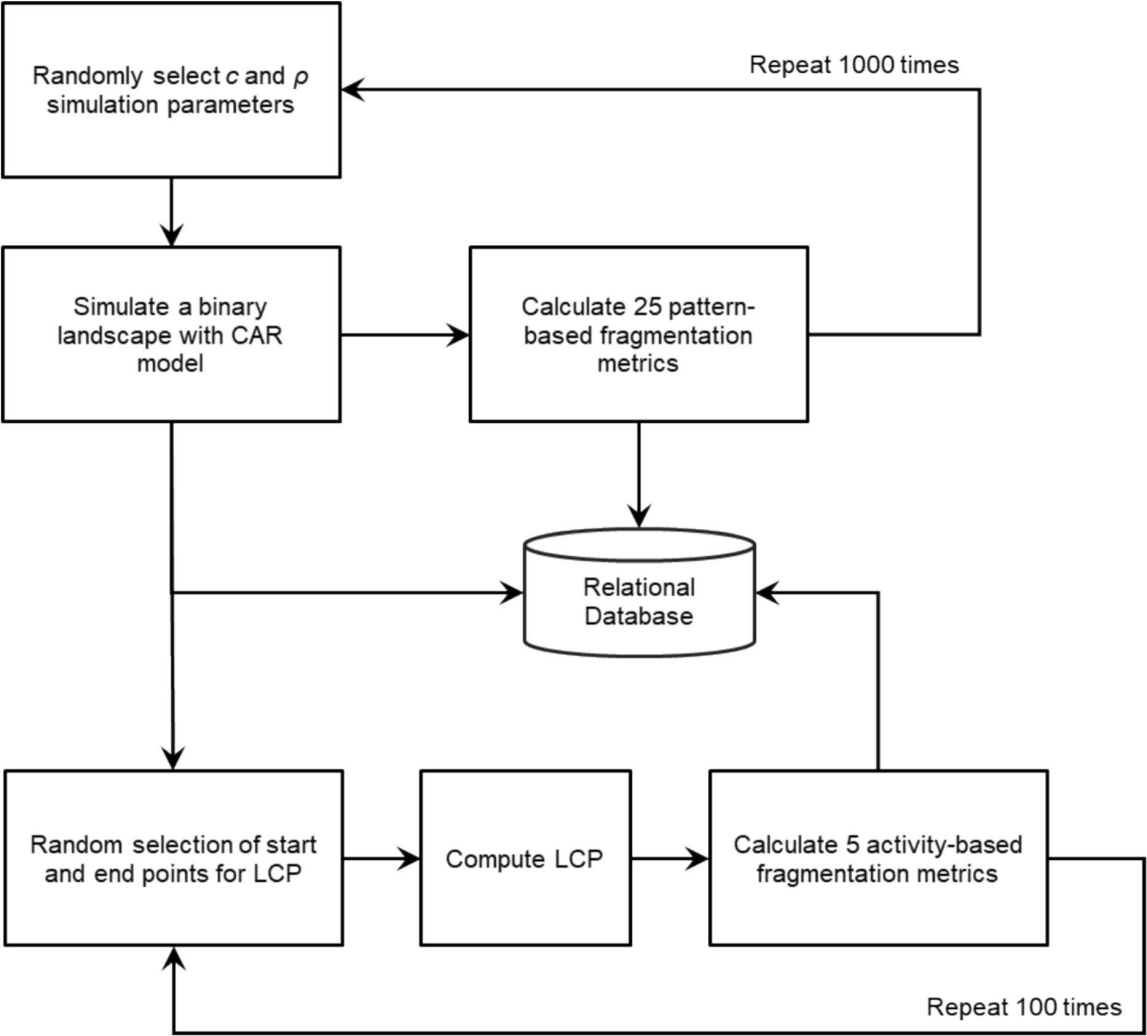
Overview of the research design and workflow, where *c* is the proportion of white landscape cells, *ρ* is the spatial autocorrelation parameter, LCP is the least-cost path, and CAR is the conditional autoregressive model used to simulate landscapes

The number of steps (*Steps*) is the length (in landscape raster cells) of the LCP for the specific randomized starting and ending point combination. The cumulative cost (*TotCost*) is the sum of costs for all cells comprising the LCP. The cost-per-step (*AvgCost*) is the *TotCost* of the LCP divided by the *Steps*. The Manhattan distance (*ManDist*) provides the minimum orthogonal distance (in landscape raster cells) between the randomized start and end points and represents the theoretical shortest travel distance regardless of the cost (|*x*_1−_*x*_2_| +|* y*_1−_*y*_2_|). The distance ratio (*Ratio*) calculates the ratio between *Steps* and *ManDist*, When *Ratio* is 1, the actual LCP and *ManDist* are identical; *Ratio* increases as the LCP increasingly deviates from *ManDist*. An increasingly complex and lengthy LCP (large *Ratio*) is taken as an indicator of landscape fragmentation because a simple short route of low-cost cells cannot be navigated. After simulating our landscapes, computing the 25 pattern-based fragmentation metrics, and the 100 replicates of 5 activity-based fragmentation metrics, we compiled a database that linked all the measurements to each unique landscape and its *c* and *ρ* parameters (Fig. 2).

We individually assessed the variability and distributions of each simulation parameter and computed metric. Then, we produced pairwise correlations among all metrics, and graphed metric values relative to simulation parameters. The extensive outputs were studied to identify any metrics that had desirable properties—monotonic relationships, low variability, and an ability to separate metric values along a steep gradient of conditions. We present a selection of the more interesting results uncovered.

## Results

**Fig. 3:**
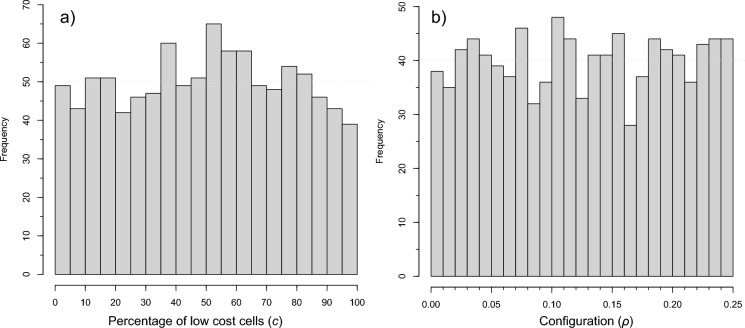
Distributions of landscape simulation parameters for **a** composition and **b** configuration

The parameters controlling the composition and configuration of our simulated landscapes were randomly drawn from uniform distributions. Evidence in Fig. 3 illustrates the nearly uniform distribution of selected parameter values within our 1000 simulations, thus confirming that all input value ranges are represented in sufficient numbers to be meaningful. While we produced distribution graphs for each individual pattern-based fragmentation metric, due to space limitations, we provide standard 5-number summaries for each metric in Table 2. The key message in this table is that some metrics are much more variable than others and that distributions are not always normal as has been emphasized previously (Remmel and Csillag 2003; Turner 2005). The precision for individual metrics varies within the table due to the scale and range at which each metric is constrained; most importantly, the distributions are computed for landscapes of consistent size, scale, and for two land cover classes. Given the consistency of landscape simulation factors, the absolute values (e.g., te: total edge) are of less significance than the relativity of computed metric values. All distribution summaries are based on calculations collected for 1000 simulated landscapes and represent the general expectation for metrics within the simulation parameter space defined.

**Table 2:** Five-number distribution summaries for the 25 pattern-based metrics computed in our study

Metric	Minimum	Median	Mean	Maximum	Std Dev
area_cv	1482	4056	3870	5980	1539.15
area_mn	1.198	2.061	3.669	12.992	3.09
area_sd	67.85	85.19	101.77	260.61	38.93
cai_cv	306.2	816.2	1871.8	6934.7	1849.99
cai_mn	0.0118	0.15717	0.19708	1.08031	0.17
cai_sd	0.739	1.412	1.483	4.239	0.56
dcad	0.01695	37.63835	27.71100	49.67584	18.48
ed	12.07	255.32	255.59	332.90	94.48
lpi	48.91	73.00	73.63	99.00	14.53
te	71190	1505925	1330602	1963500	557272
shape_cv	10.83	78.60	121.11	386.74	103.68
shape_mn	1.009	1.436	1.398	1.856	0.24
shape_sd	0.1093	1.1496	1.8279	5.8411	1.62
para_cv	4.59	18.38	16.79	24.32	4.55
para_mn	0.1073	0.1159	0.1174	0.1328	0.01
para_sd	0.006096	0.021172	0.019394	0.026136	0.00
cohesion	99.44	99.55	99.58	99.82	0.11
division	0.01992	0.46710	0.40235	0.65473	0.18
enn_cv	2.25	4.62	5.90	38.77	3.81
enn_mn	60.27	60.84	61.52	93.39	2.15
enn_sd	1.36	2.81	3.71	36.21	2.78
mesh	2036	3143	3525	5781	1064
np	454	2862	2719	4925	1524
pd	7.697	48.523	46.099	83.499	25.84
split	1.020	1.877	1.822	2.896	0.51

**Fig. 4:**
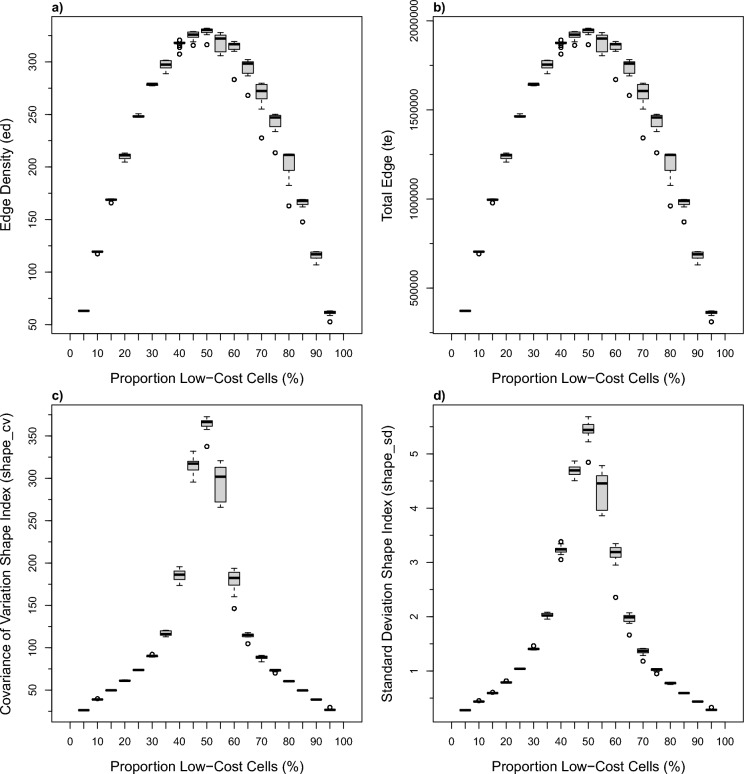
Symmetric unimodal pattern-based metrics versus proportion boxplots: **a** edge density, **b** total edge, **c** coefficient of variance of the shape index, and **d** standard deviation of the shape index

**Fig. 5:**
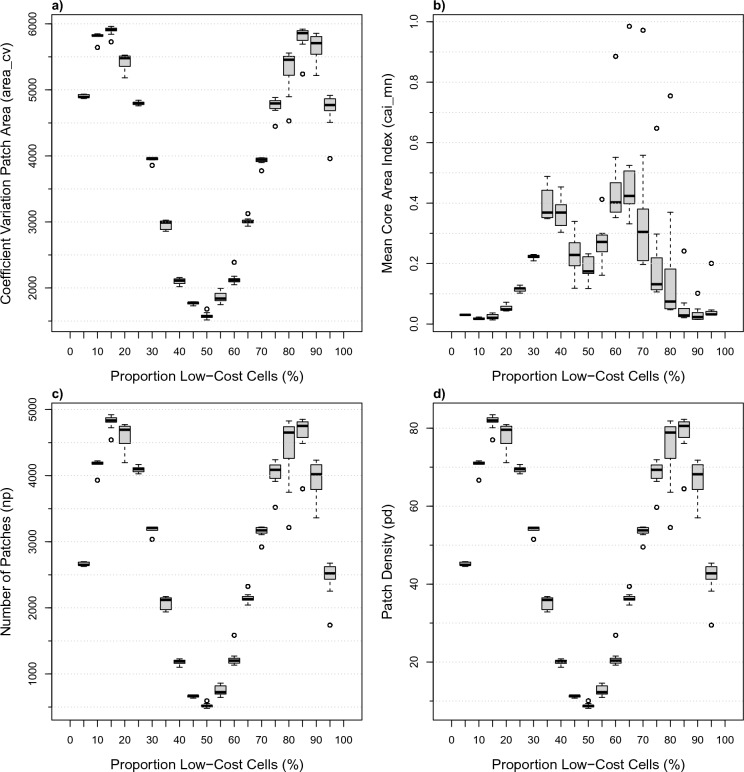
Four examples of strongly bimodal pattern-based metric distributions versus landscape composition: **a** coefficient of variation for patch area, **b** mean core area, **c** number of patches, and **d** patch density

We then plotted distributions for individual metrics relative to the proportion of low-cost cells (%), using the full set of 1000 simulations. These plots allow the expected fragmentation metric to be estimated given the proportion of white to black landscape cells. The metrics produced distributions symmetric around the 50% white cells proportion, though with distribution shapes varying among a few common forms (e.g., Fig. 4). The challenge with these pattern-based fragmentation metrics is that the same metric value is produced by 2 very different landscape compositions. This scenario gets worse (Fig. 5) where 4 different landscape compositions can produce identical metric values. The challenge then is to decide whether the landscape is fragmented in some way, or whether the proportion of white to black cells has shifted.

In addition to the lack of a one-to-one relationship between landscape composition and pattern-based fragmentation metrics, some pattern-based fragmentation metrics gave very little information regarding the level of fragmentation. For example, cohesion is a measure of aggregation that theoretically ranges from 0 to 100% and approaches zero when patches are more isolated. However, in our 1000 simulated landscapes with the proportion of white to black cells varying from 1 to 99%, all cohesion values fell within a very narrow range between 99.44% and 99.82%. Thus, there is little differentiation based on proportion and we conclude that these metrics are not effectively differentiating levels of fragmentation.

**Fig. 6:**
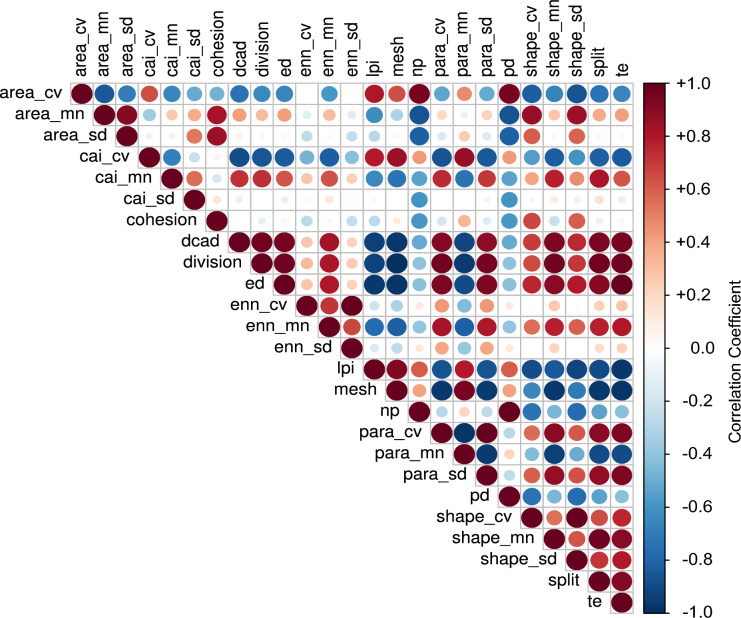
A summary of pair-wise correlation coefficients for our 25 pattern-based fragmentation metrics. The area of the circles shows the absolute correlation value, and the colour identifies the directionality of the relationship

Another criticism of patten-based metrics has been redundancy among metrics. We summarize all pair-wise correlations among our pattern-based fragmentation metrics to identify the frequency, strength, and direction of their interdependence (Fig. 6). The correlation coefficients are coloured according to their value; positive correlations are displayed in red tones and negative correlations are displayed in blue tones, with the circle size being proportional to the correlation magnitude. Lightly coloured disks indicate weak correlation. A few perfect correlations exist due to the definitions of the metrics (e.g., number of patches—*np* and patch density—*pd*). Most of the pattern metrics are correlated to some degree with other pattern metrics.

Turning our attention now toward the activity-based fragmentation metrics, for each simulated landscape we computed 100 LCP, each with random start and end points. For each of these paths, we computed the five proposed activity-based fragmentation metrics and recorded the results. The descriptive statistics for these activity-based metrics are provided in Table 3.

**Table 3:** Descriptive statistics for the activity-based fragmentation metrics

Activity-Metric	Minimum	Median	Mean	Maximum	Std Dev
*Steps*	256.0	386.0	395.3	798.0	77.11
*TotCost*	257	2676	2676	45549	9009.20
*AvgCost*	1.000	5.707	20.410	99.236	25.87
*ManDist*	256	331.0	340.9	511.0	60.20
*Ratio*	1.000	1.086	1.172	2.661	0.21

**Fig. 7:**
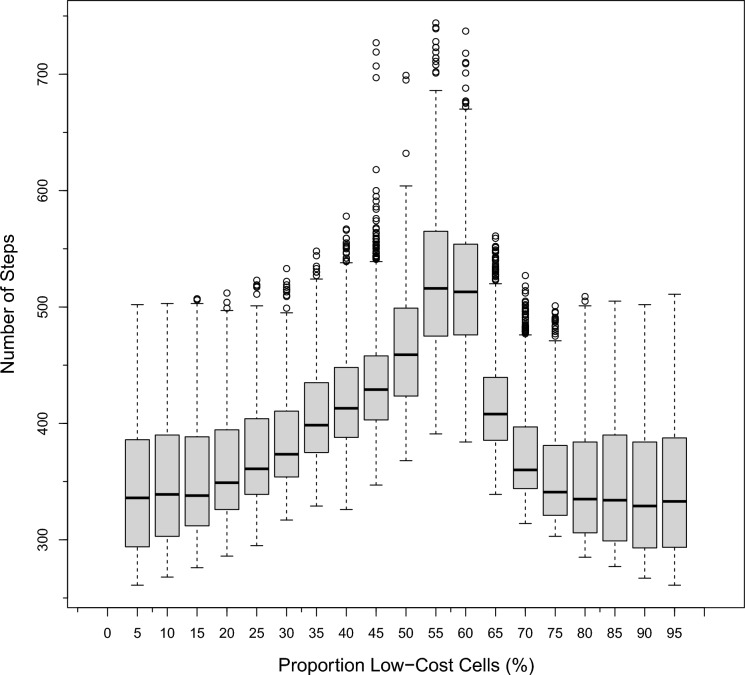
Number of steps (*Steps*) versus the proportion of low-cost cells (%)

The activity-based fragmentation metrics took on a slightly different distribution when computed for the same landscapes as the pattern-based fragmentation metrics. Rather than being symmetric around the 50% land cover proportion parameter, some metrics seemed to peak at about 59% low-cost cells (e.g., Fig. 7). In the presented figure, where most cells are costly to cross (left half of the *x*-axis), the number of steps will be low since you need to cross expensive cells anyway, so you can travel more directly. As low-cost cells increase, the number of steps will go up as those cells are incorporated into the path to reduce costs, but at the same time, increasing the length of each path. Once the number of low-cost cells breaches the 59% level, straighter paths are again possible, and the number of steps decrease.

**Fig. 8:**
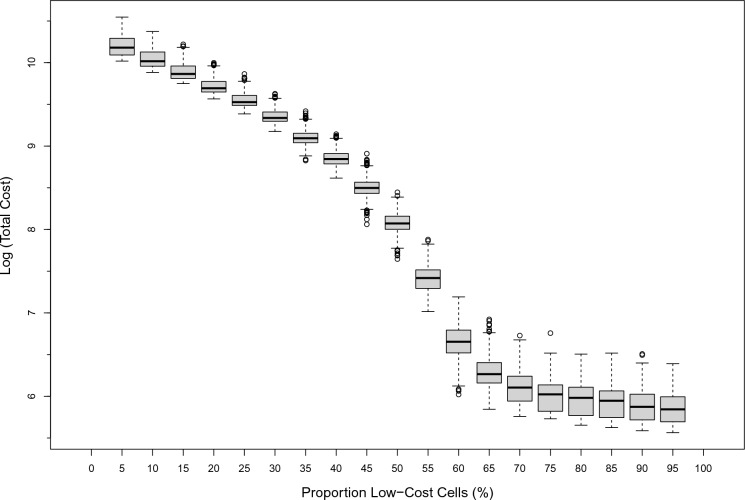
The log-scale of total cost (*TotCost*) to traverse the landscape versus the proportion of low-cost-cells (%)

**Fig. 9:**
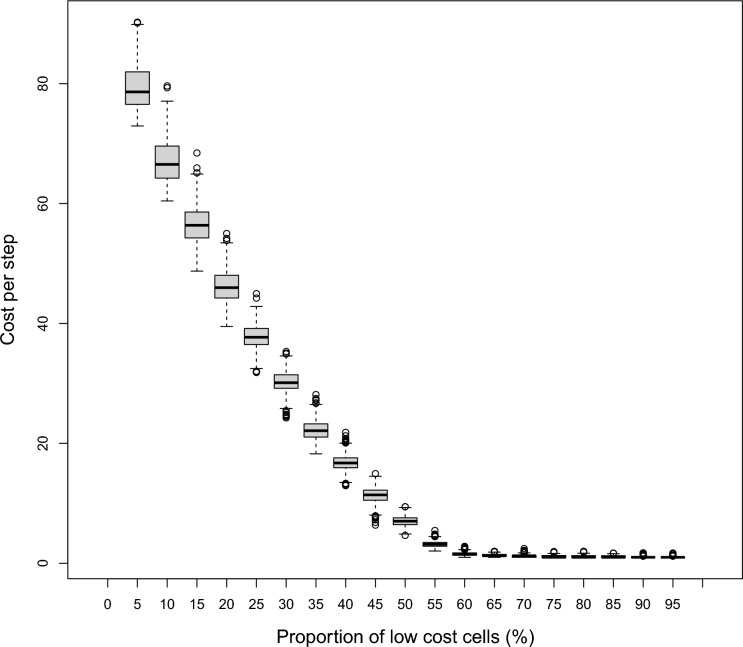
The cost-per-step (*AvgCost*) versus proportion of low-cost cells (%)

In addition to looking at the length of paths, we also summarize the total cost of paths (*TotCost*) dependent on the number of low-cost cells comprising the landscape (Fig. 8). This summary presents a monotonic function that is much more useful than the symmetric (or quasi-symmetric) distributions that we have already seen, since here, significant differences in landscape composition cannot produce an identical result to the fragmentation metric. Figure 8 illustrates that the proportion of land cover classes has a profound effect on the cost to cross landscapes and that this metric provides an indication of fragmentation that incorporates the underlying landscape composition and the activity being measured on the landscape simultaneously (Table 4). As the proportion of low-cost cells increase, overall costs decrease due to the ability to avoid high-cost cells; this is supported by the average cost-per-step (Fig. 9).

**Table 4:** Correlations between pattern-based (P) and activity-based (A) fragmentation metrics and landscape simulation parameters

Metric	Type	*r*: c	r: *ρ*
area_cv	P	− 0.034	− 0.017
area_mn	P	0.014	− 0.021
area_sd	P	0.022	− 0.016
cai_cv	P	− **0.129**	− **0.132**
cai_mn	P	**0.104**	**0.249**
cai_sd	P	**0.142**	**0.244**
dcad	P	0.011	0.001
ed	P	− 0.003	− 0.027
lpi	P	0.011	− 0.018
te	P	− 0.003	− 0.027
shape_cv	P	− 0.001	− 0.017
shape_mn	P	0.006	− 0.027
shape_sd	P	− 0.002	− 0.029
para_cv	P	**0.104**	− **0.157**
para_mn	P	− 0.076	− 0.107
para_sd	P	**0.111**	**0.163**
cohesion	P	− 0.009	− 0.015
division	P	0.016	0.021
enn_cv	P	**0.169**	**0.256**
enn_mn	P	**0.098**	**0.130**
enn_sd	P	**0.172**	**0.256**
mesh	P	− 0.016	− 0.021
np	P	− 0.034	− 0.020
pd	P	− 0.034	− 0.020
split	P	0.015	0.014
*Steps*	A	0.00	0.024
*TotCost*	A	− **0.90**	− 0.035
*AvgCost*	A	− **0.88**	0.035
*ManDist*	A	− 0.03	0.016
*Ratio*	A	0.01	0.022

Significant *r* values (α = 0.01) are bolded

The relationship in Fig. 8, when presented in non-logarithmic form, can be split into two functions. The first is a quadradic function for the descending limb of *TotCost* between 1 and 59% proportion of low-cost landscape cells ($y=5.05{x}^{2}-810.68x+31103.71; {r}^{2}=0.998$). The second function is for the remaining range of low-cost cell proportions and can be explained by a linear function ($y=-7.99x+1070.79; {r}^{2}=0.696$).

The LCP distance relative to the corresponding Manhattan distance (*Ratio*) produced a distribution like that observed in Fig. 7 which peaks at about 59% low-cost cells. On its own, this is not as useful as we had hoped, given that it provides a comparison of the actual LCP to the theoretical shortest distance between start and end points. This *Ratio*, while not perfect, does however indicate the additional path length required to traverse landscapes relative to the underlying composition (unfortunately identical *Ratio* values can be obtained by approximately inverse proportions of our two land cover classes. Regardless, this deviation from unity provides an idea of how complex the landscape might be and when considered in conjunction with *Steps*, *TotCost*, and *AvgCost*, a fuller picture of a landscape’s fragmentation state becomes possible.

## Discussion

Models can be useful in landscape studies because it is generally difficult, if not impossible, to perform experiments at a desired spatial or temporal scale (Turner et al. 1989). Our implementation allows for flexible implementation but also for assessing the distributions of outcomes. With real landscapes, our number of samples would be much too small to fully assess metric behaviour. Our use of LCP as a landscape activity originates from geographic transportation literature (Diniz et al. 2020) and establishes connectivity between two locations that is more ecologically realistic than using Euclidean distance because the LCP takes into account the perceived ecological costs of traversing a landscape (Etherington and Holland 2013). Results from LCP analyses provide not only the accumulated cost, but also a map of the path, making results inherently spatial (Savary et al. 2022). While our selection of the LCP is a relatively simplistic activity measure, it captures elements of terrain, animal psychology, metabolism, risk, energy expenditure, landscape connectivity, and corridor identification (Zeller et al. 2012; Alexander et al. 2016) in assessing activity on fragmented landscapes (Sawyer et al. 2011; Correa Ayram et al. 2016; Etherington 2016) and thus is suitable for our high-level examples.

We would like to emphasize the flexibility of our framework, in that any activity could be simulated on these landscapes, and that any metrics could be designed or computed to characterize those activities. Our use of the LCP and its derivative summary metrics was due to its intuitive and simple implementation; however, with deeper knowledge of specific landscapes, organisms, and their behaviour, all of these aspects of the implementation can be modified. Our contribution highlights that the fragmentation of a landscape can affect the activity that unfolds on it.

An area of dispute in the conservation research community is known as the single large or several small debate (SLOSS—Single Large Or Several Small) regarding the relative value of large reserves versus collections of small reserves that protect the same total area (Fahrig et al. 2022). In the mid-1970s, environmental conservationists proposed that when designing nature reserves, for a fixed total area, a single large reserve will preserve more species that several smaller ones. Prioritizing the conservation of large patches over small patches has been a practice throughout the world (Lindenmayer 2019; Fahrig 2020; Riva and Fahrig 2022) and may have had the unintended consequence of underrating the importance of small remnant patches in fragmented landscapes (Wintle et al. 2019). The extinction risk has, however, been observed as higher in small patches because smaller populations are more susceptible to environmental stochasticity (Laurance 2002; Riva and Fahrig 2022). Recently, it has been recognized that both single large and many small fragments are needed to promote landscape-wide biodiversity (Riva and Fahrig 2023). Thus, the quantification and monitoring of landscape fragmentation is of interest to ecologists or wildlife and resource managers with often competing needs.

Landscape ecology studies the reciprocal interactions that occur between spatial pattern and ecological processes (Rutledge 2003; Turner 2005). For example, changes in landscape structures can affect the distribution, movement, and persistence of some species (Turner 1989), population dynamics, biogeochemical cycling, biodiversity (Mas et al. 2010), and surface water runoff (Krummel et al. 1987). Maintaining connectivity is seen as an important goal for biodiversity conservation worldwide (Ribeiro et al. 2017). It has been found that extinction cascades are more likely to occur in landscapes with low landscape connectivity and low or degraded native vegetation cover.

Landscape composition can influence landscape configuration and functional connectivity. For example, in landscapes with low forest cover, nearest-neighbour forest patches were often found to be distant from one another, have a large number of patches and increased edge with some interpatch distances being quite large for the three forest birds under study (Bélisle et al. 2001). Thus, maintaining and restoring connectivity among habitat patches is seen as an effective conservation practice for facilitating species movement, gene flow, and increased population persistence in fragmented landscapes (Dutta et al. 2022). Corridors within a landscape can increase the movement and genetic exchange between populations in different habitat patches and possibly save populations from extinction (Alexander et al. 2016). Connectivity has also been used to evaluate and model the spread of invasive species (Helfenstein et al. 2014). Hence, having robust methods for quantifying fragmentation are of paramount importance.

Interestingly percolation theory, which relates the connectiveness of the matrix to the proportion of the landscape occupied by patches by defining when a matrix inversion occurs, relates to some of the results that we discovered. Using the rook’s case connectivity (Luo et al. 2018), a critical proportion of the focal class probability occurs where the largest cluster will connect the map continuously from one side to the other occurs at 0.5928 (Gardner et al. 1987), for very large arrays. Our results, based on landscapes produced using a stationary conditional autoregressive simulator produced metric results in-line with this value drawn from percolation theory. Many of our metrics produced expected peak values or points at which relationships changed at this land cover proportion threshold (e.g., *TotCost*, *AvgCost*).

Results from a sequence of neutral modelling studies indicate that the amount of habitat (i.e., the composition) is the single best predictor of landscape pattern (Gardner and Urban 2007) and studies have also shown that human vision is more sensitive to changes in composition than configuration (Boots and Csillag 2006). While both composition and configuration parameters are required to characterize or simulate a binary landscape, the composition parameter is often influential on measured dependent metrics, whereas the more subtle configuration parameter showed no effect. This makes sense since composition radically influences the land cover present, whereas configuration simply modifies how the classes cluster. It is therefore the abundance of low-cost cells that more dramatically influences our activity metrics, rather than how tightly they cluster on the landscape. Furthermore, given the stationarity and isotropy of our landscape simulator, the directionality of our simulated activity (LCP) has no impact metrics that summarize it. For real-world landscapes however, some omnidirectional metrics should be considered.

All of this development and testing culminates into the question of what should come next? While our implementation provides the framework for using a proxy (landscape activity) to make inference regarding the state of landscape fragmentation, it does so in a highly theoretical sense. We foresee this framework as being highly modular, where the landscape simulator could be changed or swapped out for real landscapes. We expect that different activities could be substituted for our LCP implementation, and that derivative metrics pertaining to that activity could also be tailored to measure specifics related to an organism, activity, or scenario of interest. This implies working with ecologists and biologists on highly specific case studies. We also expect that from a theoretical perspective, different landscape simulators (particularly those that produce non-stationary and non-isotropic patterns) could yield new insights and leverage omni-directional activity characterization metrics. Once organism/activity specifics are identified, simulations could build baseline and expectation datasets to which empirical comparisons of real landscapes could be made.

## Conclusions

We tested the relative improvements of activity-based metrics for quantifying landscape fragmentation. We measured the sensitivity of this activity-based approach for capturing subtle fragmentation effects and then compared it against existing pattern-based approaches of characterizing fragmentation. The goal was to test for potential benefits of a quantification metric that is based on a simulated stochastic process acting on the landscape to arrive at a quantification, rather than a static metric based purely on composition and configuration. One thousand simulated landscapes, varying in composition and configuration, provided the study data. One-hundred LCP with random start and end points along opposing landscape margins were determined for each of the 1000 simulated landscapes. Various ways to characterize the cost to traverse a landscape became our proxy measurements for quantifying landscape fragmentation.

This new approach of developing an activity-based fragmentation quantification method yielded a measure that was more sensitive to landscape fragmentation than a pattern-based quantification. Specifically, two of the variables, cumulative cost (*TotCost*) and the derived value cost-per-step (*AvgCost*), are strongly related to landscape composition with a monotonic relationship. As expected, neither the pattern-based nor the activity-based metrics were significantly sensitive to the landscape simulation parameter for configuration, but the activity-based metrics are influenced by the spatial arrangement of land cover types. The approximate threshold of 59% for low-cost land cover cells on a landscape mirror that which is postulated by percolation theory and remains of critical importance when observing the functional forms of metric values relative to land cover proportions.

This activity-based method overcame the issue of scale by holding the extent and grain of the landscape constant over 1000 simulated landscapes. It can easily be implemented and modified through readily available free open-source software, allowing other activities to be implemented and quantified as needs dictate. Our study provides a methodological foundation for further studies as this activity-based approach is tested for specific environments and species assemblages.

## Data Availability

No datasets were generated or analysed during the current study.

## References

[CR1] Alexander JL, Olimb SK, Bly KLS, Restani M (2016) Use of least-cost path analysis to identify potential movement corridors of swift foxes in Montana. JMAMMAL 97:891–898

[CR2] Antrop M (2021) Landscape mosaics and the patch-corridor-matrix model. The Routledge handbook of landscape ecology, 1st edn. Routledge, London, pp 25–48

[CR3] Baker W, Cai Y (1992) The r.le programs for multiscale analysis of landscape structure using the GRASS geographical information system. Landscape Ecol 7:291–302

[CR4] Bélisle M, Desrochers A, Fortin M-J (2001) Influence of forest cover on the movements of forest birds: a homing experiment. Ecology 82:1893–1904

[CR5] Bogaert J (2003) Lack of agreement on fragmentation metrics blurs correspondence between fragmentation experiments and predicted effects. Conserv Ecol. 10.5751/ES-00495-0701r06

[CR6] Boots B, Csillag F (2006) Categorical maps, comparisons, and confidence. J Geogr Syst 8:109–118

[CR7] Correa Ayram CA, Mendoza ME, Etter A, Salicrup DRP (2016) Habitat connectivity in biodiversity conservation: a review of recent studies and applications. Prog Phys Geogr: Earth Environ 40:7–37

[CR8] Costanza JK, Riitters K, Vogt P, Wickham J (2019) Describing and analyzing landscape patterns: where are we now, and where are we going? Landscape Ecol 34:2049–2055

[CR9] Cushman SA, McGarigal K, Neel MC (2008) Parsimony in landscape metrics: strength, universality, and consistency. Ecol Ind 8:691–703

[CR10] Diniz MF, Cushman SA, Machado RB, De Marco JP (2020) Landscape connectivity modeling from the perspective of animal dispersal. Landsc Ecol 35:41–58

[CR11] Driscoll DA, Armenteras D, Bennett AF et al (2021) How fire interacts with habitat loss and fragmentation. Biol Rev 96:976–998 33561321 10.1111/brv.12687

[CR12] Dutta T, Sharma S, Meyer NFV et al (2022) An overview of computational tools for preparing, constructing and using resistance surfaces in connectivity research. Landsc Ecol 37:2195–2224

[CR13] Etherington TR (2016) Least-cost modelling and landscape ecology: concepts, applications, and opportunities. Curr Landscpe Ecol Rep 1:40–53

[CR14] Etherington TR, Holland EP (2013) Least-cost path length versus accumulated-cost as connectivity measures. Landsc Ecol 28:1223–1229

[CR15] Fahrig L (2003) Effects of habitat fragmentation on biodiversity. Annu Rev Ecol Evol Syst 34:487–515

[CR16] Fahrig L (2017) Ecological responses to habitat fragmentation per se. Annu Rev Ecol Evol Syst 48:1–23

[CR17] Fahrig L (2019) Habitat fragmentation: a long and tangled tale. Global Ecol Biogeogr 28:33–41

[CR18] Fahrig L (2020) Why do several small patches hold more species than few large patches? Global Ecol Biogeogr 29:615–628

[CR19] Fahrig L, Watling JI, Arnillas CA et al (2022) Resolving the SLOSS dilemma for biodiversity conservation: a research agenda. Biol Rev 97:99 34453405 10.1111/brv.12792 PMC9290967

[CR20] Fletcher RJ, Didham RK, Banks-Leite C et al (2018) Is habitat fragmentation good for biodiversity? Biol Cons 226:9–15

[CR21] Forman RTT (1995) Some general principles of landscape and regional ecology. Landscape Ecol 10:133–142

[CR22] Forman RTT, Godron M (1986) Landscape ecology. Wiley, New York

[CR23] Gardner RH, Urban DL (2007) Neutral models for testing landscape hypotheses. Landscape Ecol 22:15–29

[CR24] Gardner RH, Milne BT, Turner MG, O’Neill RV (1987) Neutral models for the analysis of broad-scale landscape pattern. Landsc Ecol 1:19–28

[CR25] Gustafson EJ (1998) Quantifying landscape spatial pattern: what is the state of the art? Ecosystems 1:143–156

[CR26] Haddad NM, Brudvig LA, Clobert J et al (2015) Habitat fragmentation and its lasting impact on Earth’s ecosystems. Sci Adv. 10.1126/sciadv.1500052 26601154 10.1126/sciadv.1500052 PMC4643828

[CR27] Hadley AS, Betts MG (2016) Refocusing habitat fragmentation research using lessons from the last decade. Curr Landscape Ecol Rep 1:55–66

[CR28] Haines-Young R, Chopping M (1996) Quantifying landscape structure: a review of landscape indices and their application to forested landscapes. Prog Phys Geogr 20:418–445

[CR29] Helfenstein J, Bauer L, Clalüna A et al (2014) Landscape ecology meets landscape science. Landsc Ecol 29:1109–1113

[CR30] Hesselbarth MHK, Sciaini M, With KA et al (2019) landscapemetrics: an open-source R tool to calculate landscape metrics. Ecography 42:1648–1657

[CR31] Hobbs RJ (1993) Effects of landscape fragmentation on ecosystem processes in the Western Australian wheatbelt. Biol Cons 64:193–201

[CR32] Jaeger JAG (2000) Landscape division, splitting index, and effective mesh size: new measures of landscape fragmentation. Landscape Ecol 15:115–130

[CR33] Krummel JR, Gardner RH, Sugihara G et al (1987) Landscape patterns in a disturbed environment. Oikos 48:321–324

[CR34] Kupfer JA (2012) Landscape ecology and biogeography: Rethinking landscape metrics in a post-FRAGSTATS landscape. Prog Phys Geogr 36:400–420

[CR35] Kupfer JA, Franklin SB (2009) Linking spatial pattern and ecological responses in human-modified landscapes: the effects of deforestation and forest fragmentation on biodiversity. Geogr Compass 3:1331–1355

[CR36] Laurance WF (2002) Hyperdynamism in fragmented habitats. J Veg Sci 13:595–602

[CR37] Laurance WF (2008) Theory meets reality: How habitat fragmentation research has transcended island biogeographic theory. Biol Cons 141:1731–1744

[CR38] Lausch A, Blaschke T, Haase D et al (2015) Understanding and quantifying landscape structure—a review on relevant process characteristics, data models and landscape metrics. Ecol Model 295:31–41

[CR39] Lewis J (2022) R package Least Cost Path: methods for modelling movement in the landscape. https://cran.microsoft.com/snapshot/2020-04-20/web/packages/leastcostpath/vignettes/leastcostpath-1.html. Accessed 9 Mar 2023

[CR40] Lindenmayer D (2019) Small patches make critical contributions to biodiversity conservation. Proc Natl Acad Sci USA 116:717–719 30591563 10.1073/pnas.1820169116 PMC6338845

[CR41] Luo Q, Griffith DA, Wu H (2018) On the statistical distribution of the nonzero spatial autocorrelation parameter in a simultaneous autoregressive model. IJGI 7:476

[CR42] Mas J-F, Gao Y, Pacheco JAN (2010) Sensitivity of landscape pattern metrics to classification approaches. For Ecol Manag 259:1215–1224

[CR43] McGarigal K, Marks BJ (1995) FRAGSTATS: spatial pattern analysis program for quantifying landscape structure. Gen Tech Rep PNW-GTR-351 Portland, OR: US Department of Agriculture, Forest Service, Pacific Northwest Research Station 122 p 351

[CR44] McGarigal K, Cushman S, Regan C (2005) Quantifying terrestrial habitat loss and fragmentation: a protocol. Department of natural resources conservation, University of Massachusetts

[CR45] Neel MC, McGarigal K, Cushman SA (2004) Behavior of class-level landscape metrics across gradients of class aggregation and area. Landsc Ecol 19:435–455

[CR46] Pfeifer M, Lefebvre V, Peres CA et al (2017) Creation of forest edges has a global impact on forest vertebrates. Nature 551:187–191 29088701 10.1038/nature24457 PMC5681864

[CR47] Qi Y, Wu J (1996) Effects of changing spatial resolution on the results of landscape pattern analysis using spatial autocorrelation indices. Landsc Ecol 11:39–49

[CR48] R Core Team (2021) R: A language and environment for statistical computing

[CR49] Remmel TK, Csillag F (2003) When are two landscape pattern indices significantly different? J Geogr Syst 5:331–351

[CR50] Remmel TK, Fortin M-J (2013) Categorical, class-focused map patterns: characterization and comparison. Landsc Ecol 28:1587–1599

[CR51] Ribeiro JW, Silveira dos Santos J, Dodonov P et al (2017) LandScape corridors (lscorridors): a new software package for modelling ecological corridors based on landscape patterns and species requirements. Methods Ecol Evol 8:1425–1432

[CR52] Riitters K (2019) Pattern metrics for a transdisciplinary landscape ecology. Landscape Ecol 34:2057–2063

[CR53] Riitters KH, O’Neill RV, Hunsaker CT et al (1995) A factor analysis of landscape pattern and structure metrics. Landscape Ecol 10:23–39

[CR54] Riva F, Fahrig L (2022) The disproportionately high value of small patches for biodiversity conservation. Conserv Lett. 10.1111/conl.12881

[CR55] Riva F, Fahrig L (2023) Landscape-scale habitat fragmentation is positively related to biodiversity, despite patch-scale ecosystem decay. Ecol Lett 26:268–277 36468190 10.1111/ele.14145

[CR56] Rutledge D (2003) Landscape indices as measures of the effects of fragmentation: can pattern reflect process? Dept of Conservation, Wellington

[CR57] Saura S, Martinez-Millan J (2001) Sensitivity of landscape pattern metrics to map spatial extent. Photogrammetric Engineering p. 10

[CR58] Savary P, Foltête JC, Garnier S (2022) Cost distances and least cost paths respond differently to cost scenario variations: a sensitivity analysis of ecological connectivity modeling. Int J Geogr Inf Sci 36:1652–1676

[CR59] Sawyer SC, Epps CW, Brashares JS (2011) Placing linkages among fragmented habitats: do least-cost models reflect how animals use landscapes?: Least-cost modelling for habitat linkage design. J Appl Ecol 48:668–678

[CR60] Šímová P, Gdulová K (2012) Landscape indices behavior: a review of scale effects. Appl Geogr 34:385–394

[CR61] Tischendorf L, Fahrig L (2000) How should we measure landscape connectivity? Landscape Ecol 15:633–641

[CR62] Turner M (1989) Landscape ecology: the effect of pattern on process. Annu Rev Ecol Syst 20:27

[CR63] Turner MG (2005) Landscape ecology: what Is the state of the science? Annu Rev Ecol Evol Syst 36:319–344

[CR64] Turner M, Gardner RH (eds) (2015) Landscape ecology in theory and practice. Springer, New York

[CR65] Turner M, O’Neill R, Gardner R, Milne B (1989) Effects of changing spatial scale on the analysis of landscape pattern. Landsc Ecol 3:153–162

[CR66] Uuemaa E, Antrop M, Roosaare J et al (2009) Landscape metrics and indices: an overview of their use in landscape research. Living Rev Landsc Res 3:1–28

[CR67] Walz U, Syrbe R-U (2013) Linking landscape structure and biodiversity. Ecol Ind 31:1–5

[CR68] Wang X, Blanchet FG, Koper N (2014) Measuring habitat fragmentation: an evaluation of landscape pattern metrics. Methods Ecol Evol 5:634–646

[CR69] Wickham J, Riitters KH (2019) Influence of high-resolution data on the assessment of forest fragmentation. Landsc Ecol 34:2169–2182 32076363 10.1007/s10980-019-00820-z PMC7029708

[CR70] Wiens JA (2008) Allerton Park 1983: the beginnings of a paradigm for landscape ecology? Landsc Ecol 23:125–128

[CR71] Wintle BA, Kujala H, Whitehead A et al (2019) Global synthesis of conservation studies reveals the importance of small habitat patches for biodiversity. Proc Natl Acad Sci USA 116:909–914 30530660 10.1073/pnas.1813051115 PMC6338828

[CR72] With KA (2002) The landscape ecology of invasive spread. Conserv Biol 16:1192–1203

[CR73] Wu J (2004) Effects of changing scale on landscape pattern analysis: scaling relations. Landsc Ecol 19:125–138

[CR74] Zeller KA, McGarigal K, Whiteley AR (2012) Estimating landscape resistance to movement: a review. Landsc Ecol 27:777

